# From SELEX to sequencing: uncovering enriched aptamers with next-generation sequencing and bioinformatics

**DOI:** 10.1093/bioinformatics/btag777

**Published:** 2026-10-22

**Authors:** Melis Güler Girbas, Jennifer Müller, Christian Schlensak, Meltem Avci-Adali

**Affiliations:** Department of Thoracic and Cardiovascular Surgery, University Hospital Tuebingen, Tuebingen 72076, Germany; NGS Competence Center Tuebingen (NCCT), Tuebingen 72076, Germany; Department of Thoracic and Cardiovascular Surgery, University Hospital Tuebingen, Tuebingen 72076, Germany; Department of Thoracic and Cardiovascular Surgery, University Hospital Tuebingen, Tuebingen 72076, Germany

## Abstract

**Motivation:**

The combinatorial chemistry process called systematic evolution of ligands by exponential enrichment (SELEX) enables the selection of aptamers against different targets. The integration of next-generation sequencing (NGS) into SELEX workflows represents a powerful strategy to enhance the identification and prioritization of target-binding aptamers. In this study, we present an optimized computational approach for the NGS-based analysis of enriched aptamer pools generated through SELEX. It encompasses sequencing library preparation, sequence data preprocessing, clustering, multiple sequence alignment, phylogenetic analysis, and secondary structure prediction.

**Results:**

To identify target-specific aptamers, two comparative analysis strategies were implemented. In the first strategy, sequences detected in the negative selection pool were removed from the positive selection pool to eliminate nonspecific binders, resulting in six distinct sequence clusters. In the second approach, all sequences were retained, and cluster distributions were compared between the positive and negative selection pools, identifying seven clusters in the positive and six in the negative pool. Phylogenetic relationships and structural predictions enabled the selection of promising candidates, which were prioritized based on secondary structure, sequence abundance, and genetic distance from nonspecific binders. Binding specificity was assessed through dot blot assays, confirming that the selected aptamers exhibited strong specific binding to the target protein. These results demonstrate the effectiveness of the NGS-guided workflow for aptamer discovery and highlight its value in streamlining candidate selection for downstream functional validation.

**Availability and Implementation:**

The data underlying this article will be shared on reasonable request to the corresponding author.

## Graphical abstract

**Graphical Abstract:**
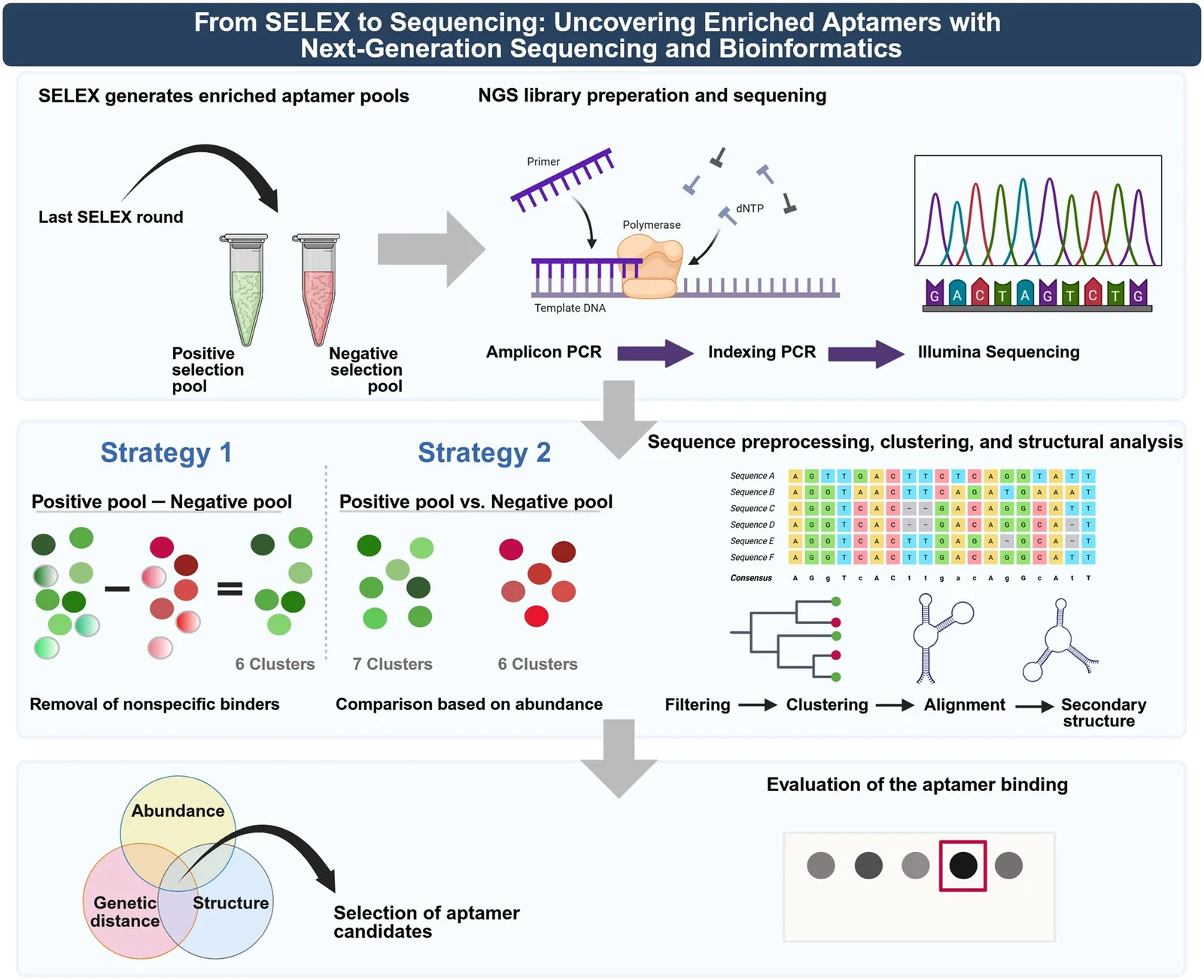

## 1 Introduction

Aptamers are single-stranded DNA (ssDNA) or RNA molecules that adopt tertiary structures, allowing them to bind to specific target molecules with remarkable affinity and selectivity (Saito 2021). They can be used for various applications, such as biosensors, diagnostics, bioimaging, drug delivery systems, and therapeutics (Futane *et al.* 2023, He *et al.* 2023, Zheng *et al.* 2025). As a favored alternative to antibodies, aptamers offer superior attributes in terms of stability and cost-effectiveness. They can be manufactured in a large scale with minimal variability between batches and a fast production time (Mohammadinejad *et al.* 2024, Wen *et al.* 2024, Zhu *et al.* 2024). The vast majority of aptamers demonstrate strong affinity towards their targets, typically characterized by dissociation constants (Kd) within the micromolar to nanomolar range, a characteristic that contributes to their preferential use in various applications (Thevendran and Citartan 2022).

Aptamers are selected using the combinatorial chemistry process, systematic evolution of ligands by exponential enrichment (SELEX), which involves iterative rounds of selection and amplification to isolate and refine aptamers with targeted binding characteristics (Didarian *et al.* 2025). Specific aptamers are generally obtained after 8–20 rounds of selection (Napit *et al.* 2025). Counter-selections against negative targets are integrated into the procedure to eliminate nonspecific binders (Alkhamis *et al.* 2023). In the last selection round, the enriched aptamer pool is sequenced, and binding kinetics of selected aptamers are assessed through diverse methodologies, such as isothermal titration calorimetry, microscale thermophoresis, surface plasmon resonance, quartz crystal microbalance, and nuclear magnetic resonance spectroscopy (Daems *et al.* 2021).

Since its development, the SELEX procedure has undergone numerous modifications and improvements to increase the efficiency of aptamer selection (Kohlberger and Gadermaier 2022; Brown *et al.* 2024). Recent advancements in SELEX include e.g. the use of lambda exonuclease digestion for generating ssDNA, which outperforms previous methods like strand separation with streptavidin-coated magnetic beads (Avci-Adali *et al.* 2009). Lambda exonuclease selectively removes the undesired phosphorylated strand of double-stranded DNA (dsDNA), yielding ssDNA with higher purity and yield, thus improving SELEX efficiency.

Traditionally, to identify specific aptamers within the enriched nucleic acid pool after the final SELEX round, the nucleic acids were cloned into a bacterial host, and 30–100 clones were sequenced using the Sanger method (Komarova and Kuznetsov 2019). However, this approach limited the analysis to a small number of sequences, resulting in the identification of only a small fraction of the potential aptamer candidates. In addition, Sanger sequencing is constrained by short read lengths and lacks quantitative information on sequence abundance, making it inefficient for analyzing the large aptamer pools that remain even after SELEX enrichment. To overcome these limitations associated with Sanger sequencing-based aptamer selection, next-generation sequencing (NGS) can be integrated into the SELEX process, enabling the identification of millions of sequences and thereby improving aptamer discovery (Blind and Blank 2015). Unlike Sanger sequencing, NGS enables high-throughput parallelization, facilitating the analysis of large aptamer pools while simultaneously delivering both sequence and abundance information (Ferreira *et al.* 2021, Bracamonte 2023). The vast amount of sequence data generated through this approach has, in turn, necessitated the development of more advanced bioinformatics tools to filter extensive sequence datasets down to a manageable set of aptamer candidates for experimental validation (Thiel *et al.* 2012, Blind and Blank 2015). Consequently, computational analysis has become an integral component of the SELEX workflow. However, although NGS has become a standard tool for monitoring aptamer enrichment, the associated computational analyses vary considerably among studies (Kinghorn *et al.* 2017). Several published workflows focus primarily on sequence abundance (Liu *et al.* 2026), whereas important aspects such as transparent preprocessing, incorporation of negative selection data, phylogenetic analysis, and detailed reporting of clustering parameters are often omitted or only briefly described. As a result, reproducing these analyses remains challenging, particularly for researchers without extensive bioinformatics expertise.

To address these limitations, we present an optimized computational workflow for NGS-based analysis that provides a comprehensive approach from raw sequencing data to the identification of candidate aptamers. This workflow integrates transparent preprocessing, systematic sequence analysis, and candidate prioritization to improve the identification of target-specific aptamers and the efficient selection of the most promising sequences from the enriched aptamer pools. By applying this approach, researchers can effectively prioritize representative candidates for subsequent binding and functional assays. To demonstrate its utility, we applied the workflow to a representative SELEX experiment, demonstrating how NGS-based analysis can be systematically performed to facilitate the identification of enriched aptamers for downstream binding evaluation.

## 2 Materials and methods

### 2.1 Primer

All oligonucleotides, including the ssDNA library and primers, were obtained from Ella Biotech (Fürstenfeldbruck, Germany). The primers used for amplifying and sequencing the ssDNA are listed in Table 1.

**Table 1:** List of primers.^a^

Name	Sequences (5ʹ → 3ʹ)	**Length (bp)**
P1	GCCTGTTGTGAGCCTCCTAAC	21
P2	Phos-GGGAGACAAGAATAAGCATG	20
OF1 with Nextera adapter 7 sequence (Nextera i7 adapter)	GTCTCGTGGGCTCGGAGATGTGTATAAGAGACAGGCCTGTTGTGAGCCTCCTAAC	55
OR1 with Nextera adapter 5 sequence (Nextera i5 adapter)	TCGTCGGCAGCGTCAGATGTGTATAAGAGACAGGGGAGACAAGAATAAGCATG	53
IFP with Illumina forward primer with P7 tag for flow cell binding	CAAGCAGAAGACGGCATACGAGATNNNNNNNNNNGTCTCGTGGGCTCGG	49
IRP with Illumina reverse primer with P5 tag flow cell for flow cell binding	AATGATACGGCGACCACCGAGATCTACACNNNNNNNNNNTCGTCGGCAGCGTC	53

a Index sequences with 10Ns, which are different for each sample for multiplexing on the flow cell.

### 2.2 Preparation of systematic evolution of ligands by exponential enrichment samples for next-generation sequencing

Protein SELEX was performed using the start library (5′-GCCTGTTGTGAGCCTCCTAAC-N49-CATGCTTATTCTTGTCTCCC-3′) and the target protein according to our previous study (Çanak-Ipek *et al.* 2025). In total, 200 pmol of the ssDNA aptamer pool from the last selection round was incubated with 10 µg target protein or nontarget protein for negative selection. Unbound ssDNA molecules were removed by washing. This negative selection was performed to be able to eliminate nonspecific binders from the aptamer pool after sequencing. ssDNA molecules binding to nontarget or target protein were amplified by polymerase chain reaction (PCR). Amplification of the binding ssDNAs was performed using 1× Taq Plus DNA Polymerase Master Mix (VWR, Pennsylvania, USA) supplemented with 0.7 µM of Primer P1 and 0.7 µM of Primer P2. PCR was performed using a PCR thermocycler (Eppendorf, Hamburg, Germany) according to the following cycle protocol: an initial denaturation at 94°C for 3 min, followed by 16 cycles of denaturation at 94°C for 45 s, annealing at 61°C for 45 s, and extension at 72°C for 1 min. The reaction was completed with a final extension at 72°C for 10 min.

#### 2.2.1 Construction of sequencing library via polymerase chain reaction

To convert the dsDNA pool obtained from the SELEX process into a sequencing library, a two-step PCR consisting of amplicon PCR followed by index PCR was performed (Fig. 1). In the first step, amplicon PCR was carried out using primers OF1 and OR1 (ELLA Biotech, Fürstenfeldbruck). These primers contain the forward (P1) or reverse (P2) primer sequences of the enriched DNA pool as well as Nextera adapter i7 or Nextera adapter i5 overhang sequences. During the index PCR, the index primers (Illumina forward [IFP] and Illumina reverse primer [IRP]) of Illumina (IDT for Illumina DNA/RNA UD Indexes, Tagmentation) were used to add a 10 bp unique sequence (index) and the flow cell binding sites at both sides of the amplicon. The index is needed for multiplexing of multiple samples on the same flow cell to separate them after the sequencing bioinformatically.

**Figure 1:**
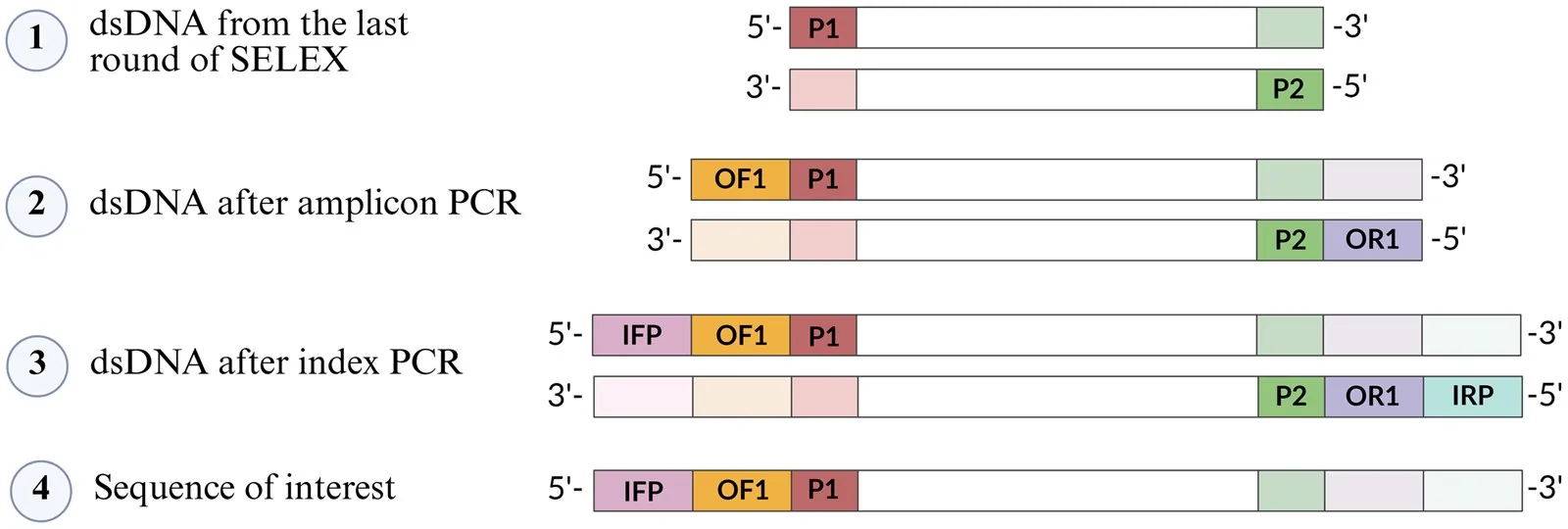
Preparation of the DNA library for sequencing using amplicon and index PCR. To prepare the DNA library for sequencing, amplicon PCR is first performed using specific primers (OF1 and OR2). During this step, a Nextera adapter overhang is incorporated into the amplicons. This is crucial to provide the binding sites for the IFP and IRP, which are used to add a 10 bp index sequence and the flow cell binding site. The 10 bp index sequence is unique for each sample and allows the multiplexing of multiple samples on the same flow cell. The resulting DNA library is then purified, quantified, and quality-checked before being loaded onto the sequencing platform.

The amplicon PCR reaction was prepared in a final volume of 2 ml containing 40 ng of dsDNA from the last selection round of SELEX, 1× Taq Plus DNA Polymerase Master Mix, 2% dimethyl sulfoxide, and 1.5 µM each of primers OF1 and OR1. PCR amplification was performed using a PCR thermocycler (Eppendorf, Hamburg, Germany) with the following conditions: an initial denaturation at 94°C for 3 min, 6 cycles of 94°C for 45 s, 61°C for 45 s, and 72°C for 60 s, followed by a final extension at 72°C for 10 min.

PCR products were purified using the MinElute PCR purification kit (Qiagen, Hilden, Germany) according to the manufacturer′s instructions. The size and quality of the PCR products were assessed using nondenaturing 5% Tris-borate-EDTA (TBE) acrylamide gel electrophoresis and 10% denaturing urea-polyacrylamide gel electrophoresis. Prior to loading onto the denaturing urea gel, samples were incubated at 95°C for 5 min. Electrophoresis was conducted with 100 ng of PCR product at 100 V for 10 min, followed by 120 V for 30 min. After electrophoresis, the gels were stained with 1x GelRed dye (Biotium, Fremont, CA, USA) for 30 min in 1× TBE buffer.

Afterwards, the sequencing library was generated via index PCR. For this, 1–3.75 µl of the purified PCR product (dependent on the concentration), 6.25 µl KAPA HiFi HotStart ReadyMix (2×) (Roche, Basel, Switzerland), and 2.5 µl index primer mix (IDT for Illumina DNA/RNA UD Indexes, Tagmentation) were mixed. Depending on the amount of purified PCR product used, the remaining volume was adjusted with H_2_O to reach a final volume of 12.5 µl. Index PCR was performed under the following thermal cycling conditions: an initial denaturation at 95°C for 3 min, followed by 8 cycles of 95°C for 30 s, 55°C for 30 s, 72°C for 30 s, with a final extension at 72°C for 5 min. Following PCR, the finished sequencing library was purified using 20 µl of AMPure XP beads and eluted in 30 µl of 10 mM Tris-HCl. The correct length of the sequencing library was verified via 2% agarose gel electrophoresis, and DNA concentration was quantified using the Qubit dsDNA BR Assay Kit (Thermo Fisher, Waltham, MA, USA). The sequencing library of all samples were pooled equimolarly based on their concentration to ensure an equal output during sequencing. Sequencing was performed on an Illumina MiSeq device (Illumina, Berlin) using a v3 sequencing kit with a 2 × 150 bp read length and sequencing depth of 1 million reads per sample.

### 2.3 Bioinformatic sequence analysis and selection of aptamer candidates

The large datasets generated by NGS were analyzed using a series of bioinformatic tools and methods. For clarity in describing the computational workflow, the following terminologies are used: Read1 represents the sequencing of the sense strand and contains the aptamer sequence in the correct orientation. Read2 represents the sequencing of the antisense strand and does not contain the aptamer sequence. However, generation of the reverse complement of Read2 yields the corresponding sense-stand sequence and thereby the correct aptamer sequence. The computational workflow (Fig. 2) consists of the following stages: basic preprocessing and adaptation of raw sequencing data, clustering of sequences within a sample, multiple sequence alignment (MSA), and secondary structure analysis of cluster representatives.

**Figure 2:**
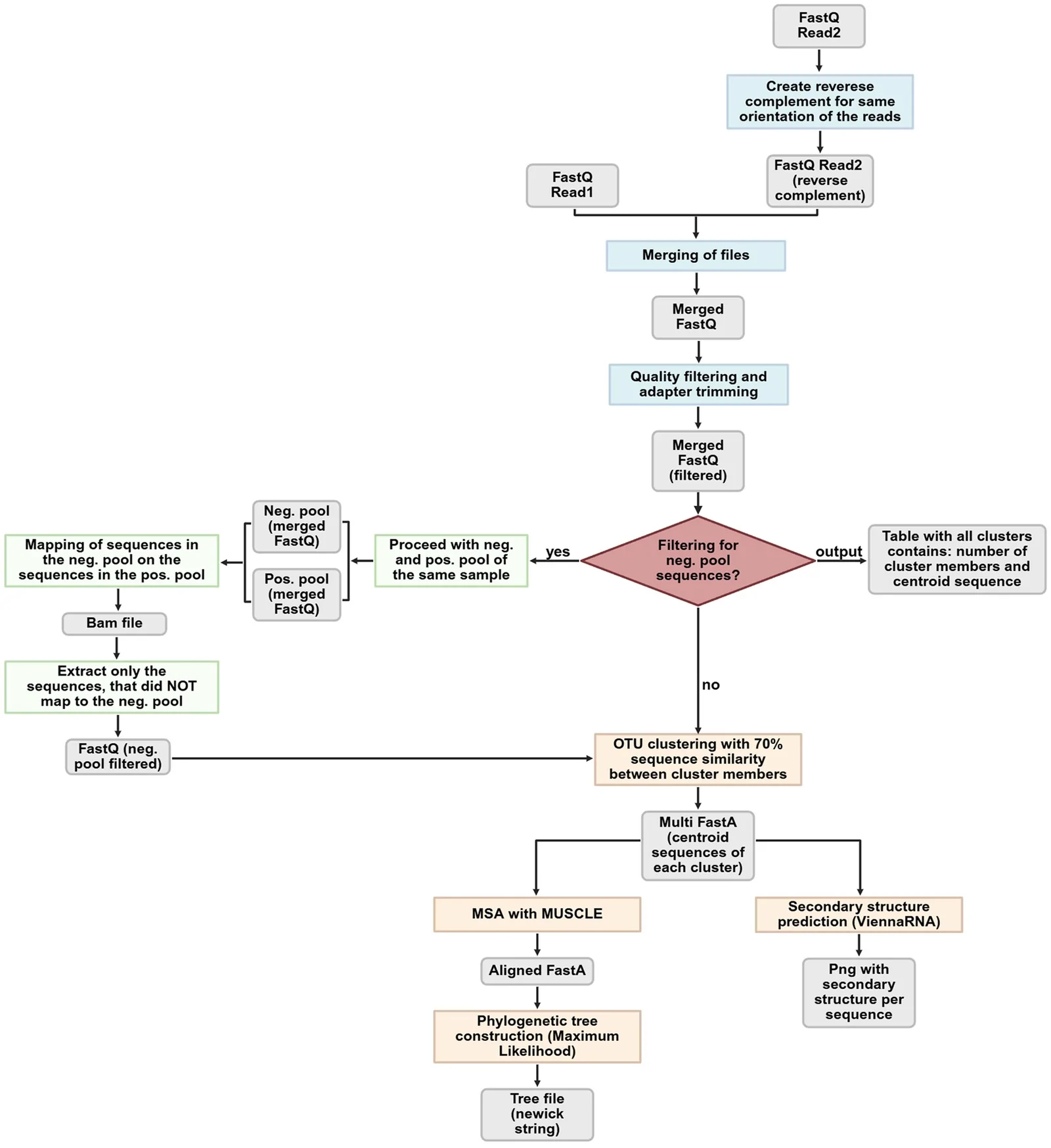
Schematic overview of the computational workflow applied for bioinformatic sequence analysis and aptamer candidate selection.

#### 2.3.1 Bioinformatic sequence analysis and selection of aptamer candidates

Sequences were demultiplexed, and FastQ files were generated for downstream analysis. A sequencing mode of 150 bp was selected to ensure complete overlap of the 90-bp-long aptamer by both reads (Read1 = sense strand = aptamer, Read2 = antisense strand = no aptamer). Since Read2 represents the sequencing of the antisense strand, which does not contain the aptamer sequence in its original orientation, its reverse complement has to be created. Thereby, the antisense-strand sequences were converted into corresponding sense-strand sequences, which contain valid aptamer sequences. This processing step leads to a duplication of the data, since both Reads contain afterwards valid aptamer sequences, which are used for further analysis steps. This increases the amount of data used for clustering and reduces bias from sequencing errors. The reverse complement of Read2 was created with *seqtk* (ver1.3), after which both FastQ files were merged into a single FastQ file (Fig. 2, blue boxes). Quality trimming and adapter removal were performed with *Trimmomatic* (ver0.39). In addition, all terminal poly-N stretches were removed (e.g. ATGGGTCNNN → ATGGGTC), and reads longer than 90 bp were trimmed to the expected aptamer length.

#### 2.3.2 Selection of the aptamer candidates from next-generation sequencing data

Two strategies were employed to identify aptamer sequences enriched against the target. In the first approach, sequences from the negative selection pool were subtracted from those in the positive selection pool (Fig. 2, green boxes). This was achieved using *bowtie2* (ver2.4.5) and *samtools* (ver1.15.1) by mapping reads to the negative control and filtering for unmapped reads to isolate sequences unique to the positive selection pool.

In the second approach, abundance differences between the negative and positive selection pools were calculated without removing sequences from the negative pool. This enabled the detection of sequences present at higher numbers in the positive pool compared to the negative pool. These sequences could also represent potential aptamers.

#### 2.3.3 Clustering of the preprocessed sequence data

For the identification of each cluster family and its enrichment, de novo operational taxonomic unit (OTU) clustering was performed using *CLC Genomics Workbench* (ver23.0.2) from Qiagen. Various sequence similarity thresholds were tested to determine the optimal balance between sequence similarity and the number of clusters. In a stepwise approach, the sequence similarity thresholds 95%, 80%, and 70% were tested. As the sequence similarity thresholds decreased, the number of clusters was reduced, with an increasing number of sequences grouped within the same cluster. Based on this evaluation, a sequence similarity threshold of 70% was selected. Each cluster was represented by its centroid sequence, which shared ≥70% sequence similarity with all other members of the same cluster and <70% similarity with sequences from other clusters. Subsequent analyses, including MSA and secondary structure prediction, were performed exclusively on these centroid sequences.

#### 2.3.4 Multiple sequence alignment with multiple sequence comparison by log-expectation

MSA was performed using MUltiple Sequence Comparison by Log-Expectation (MUSCLE, ver3.8.31) for all centroid sequences with at least 10 cluster members, demonstrating coherence among clustered sequences. The MUSCLE tool is included in the *CLC Genomics Workbench* (ver23.0.2).

#### 2.3.5 Creation of the phylogenetic tree based on multiple sequence alignment

Phylogenetic trees were constructed to visualize and analyze sequence similarities between the obtained sequences. A maximum likelihood phylogeny was calculated based on the previously generated MSA using the neighbor-joining approach (Saitou and Nei 1987) and the Jukes–Cantor substitution model (Jukes and Cantor 1969). Afterwards, bootstrapping with 1000 replicates was performed to assess the robustness of the resulting tree.

#### 2.3.6 Secondary structure prediction

For the aligned sequences representing each cluster, the secondary structures of the aptamers were predicted using the *ViennaRNAs RNAfold* (ver2.5.1) software.

#### 2.3.7 Evaluation of the binding of selected aptamers by dot blot assay

Selected aptamers were synthesized by ELLA Biotech with a biotin tag at their 5′-ends. For the dot blot assay, 10 µg of the target protein or nontarget protein were immobilized on a nitrocellulose membrane (0.45 μm, Thermo Fisher, Waltham, MA, USA). The membrane was then blocked for 1 h at room temperature with 2 ml of blocking solution phosphate-buffered saline with Mg_2_^+^ and Ca_2_^+^ (PBS^+^) containing 5% bovine serum albumin (BSA, Sigma-Aldrich, St. Louis, MO, USA) and 0.01% Tween 20 (Sigma-Aldrich) to prevent nonspecific binding. Membranes were washed with PBS^+^ and then incubated with 80 pmol biotinylated aptamers in 700 µl PBS^+^ for 1 h at 37°C. To remove unbound aptamers, the membrane was washed three times for 10 min each with PBS^+^ containing 0.2% BSA. Afterwards, the membrane was incubated for 1 h with streptavidin-HRP (Sigma-Aldrich) diluted 1:150 in PBS^+^. Next, the membrane was washed four times with PBS^+^ containing 0.01% Tween 20 and once with PBS^+^ for 5 min. Finally, 2 ml ECL Western blotting solution (Sigma-Aldrich) was applied to the membrane, incubated for 5 min, and immediately examined using the iBrightCL1000 imaging system (Invitrogen, Waltham, MA, USA). Chemiluminescence signals were quantified using *ImageJ* (ver1.54p) by measuring the mean pixel intensity of individual dots on an 8-bit grayscale scale. Measurements were performed for three independent replicates. Statistical analysis was conducted using *GraphPad PRISM software* (ver9.4.1; GraphPad Software, Inc., San Diego, USA) with one-way ANOVA followed by Tukey’s multiple comparisons test. Differences with *P* ≤ 0.05 were considered significant. Results are presented as mean + SD (*n* = 3).

## 3 Results

### 3.1 Preparation of sequencing library

*In vitro* selection of aptamers against a target protein was conducted using protein SELEX (positive selection). To eliminate nonspecific binders, the selected ssDNA pool was also incubated with a nontarget protein (negative selection). Amplicon PCR followed by index PCR was then performed with both the positive and negative selection pools. During amplicon PCR, the Nextera adapters were added. The size and purity of the amplified PCR products were assessed, and a predominant fragment with a size of ∼171 bp could be detected. Subsequently, index PCR was performed to incorporate Nextera indices at the 5′-end of the sequences, resulting in the final sequencing library. The library showed a size of 244 bp, which is the expected size for the indexed amplicon (171 bp + 34 bp P7 + 39 bp P5 = 244 bp). Thereby, P7 and P5 represent the added sequences of the 10 bp index and the flow cell binding site.

### 3.2 Next-generation sequencing and preprocessing of the sequencing data

Both constructed libraries were sequenced in 2 × 150 bp paired-end mode, which provides complete overlap across the aptamer sequence, resulting in twice the amount of data (Read1 = sense = aptamer and Read2 = antisense = no aptamer). To ensure consistent sequence orientation, the reverse complement of Read2 was generated so that both reads aligned in the same 5′→3′ direction as Read1. After this conversion, the resulting Read2 sequence corresponds to the sense strand and therefore contains a valid aptamer sequence. To preserve high data quality, minor preprocessing was applied to the raw reads, including removal of Nextera adapter and index primer as well as PCR primers. Initial quality control revealed read lengths ranging from 40 to 150 bp, with a sharp peak at 91 bp representing 85% of the sequences (Fig. 3A). Since the sequence quality declined beyond 90 bp, reads longer than 90 bp were trimmed (Fig. 3B, red line). Additionally, low-quality reads containing tailing Ns or remaining adapter sequences were removed.

**Figure 3:**
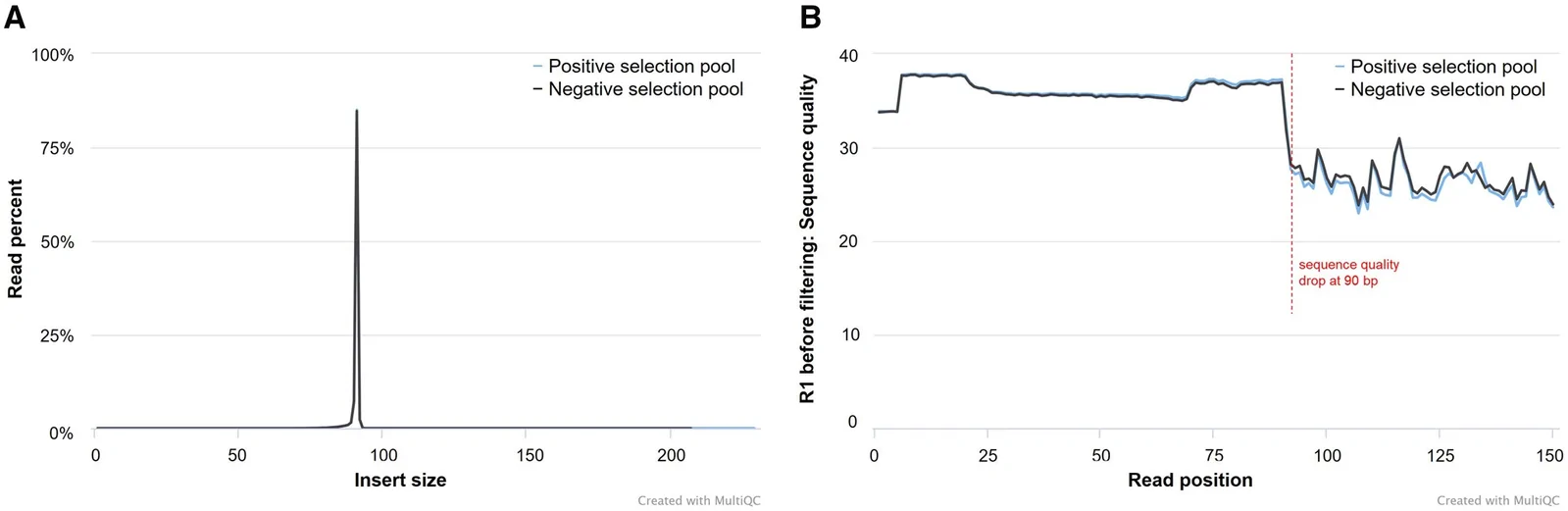
Sequence quality check of raw data generated after NGS with fastp and visualized with MultiQC. (A) The samples showed a consistent insert size of 90 bp, which was reflected by the single peak in the plot. (B) A significant drop in the sequence quality was observed at position 90. The sequence quality dropped from a Q-score of 37.27 to 28.2 (drop is marked with red dotted line).

### 3.3 Selection of aptamer sequences from the next-generation sequencing data

To identify the aptamer sequences with the most potent binding properties, two approaches were employed. The first, a highly stringent approach, aimed to minimize potential nonspecific binding to the negative control. In this method, all sequences detected in the negative selection pool (1 283 199 reads) were subtracted from those in the positive selection pool (2 447 099 reads) by mapping the reads against each other. Only reads that did not entirely map on the negative pool were used in the further analysis. This led to a substantial reduction in the number of sequences, leaving < 5% remaining (0.58%, 14.260 reads). The second, less stringent, approach included all sequences and compared the number of cluster members between the negative and positive selection pools. A high binding property for the detected sequences was assumed if a substantial difference was observed, characterized by few cluster members in the negative selection pool and a significantly higher number in the positive selection pool.

#### 3.3.1 First approach: subtracting sequences of the negative selection pool from the positive selection pool

##### 3.3.1.1 Clustering and multiple sequence alignment of preprocessed sequencing data

Sequence alignment is used to arrange two or more DNA sequences to identify regions of similarity. It is performed using algorithms that maximize matching characters while minimizing gaps or mismatches between sequences. These similarities can reveal potential relationships between sequences in terms of structural, functional, or evolutionary relationships. It plays a crucial role in various applications, such as estimating phylogenetic trees and predicting secondary structures.

Before performing MSA, OTUs were defined using a 70% sequence similarity threshold between cluster members. This threshold can be adjusted based on the amount of data and the expected similarity of the aptamers. Increasing the similarity threshold yields a greater number of clusters composed of nearly identical sequences, while decreasing it produces fewer but larger clusters.

Thereby, a file containing the representative sequences for each cluster was obtained as input for MUSCLE for the MSA creation. It is important to note that at least three valid representative sequences were required (eq. three cluster), as MUSCLE is not designed for pairwise alignments. A total of six clusters were identified, containing 46, 54, 70, 118, 128, or 315 members.

##### 3.3.1.2 Identification of secondary structures and reconstruction of the phylogenetic tree

Using ViennaRNA, the secondary structure of a representative aptamer from each cluster was predicted (Fig. 4). The numerical value associated with each aptamer candidate indicates the total count of sequences identified within the corresponding cluster. Due to their structural differences and their distant evolutionary relationship, aptamers 46 and 128 were selected for the subsequent binding analysis. The evolutionary relationships were inferred from the phylogenetic tree generated from the representative of the selected cluster. In this tree, sequences separated by greater evolutionary distance appear farther apart, which is indicated by the total path length connecting them. Each horizontal branch corresponds to an inferred common ancestor of the sequences branching from it. The length of each horizontal branch reflects the relative evolutionary distance between the sequences (e.g. between the representatives of Cluster 3 and Cluster 4 in Fig. 4). The total evolutionary distance between any two sequences is given by the sum of all branch lengths, both horizontal and vertical, along the connecting path. This structural representation allows for a clear visualization of sequence clustering, diversification patterns, and lineage relationships, thereby supporting the selection of structurally distinct aptamers for further functional characterization.

**Figure 4:**
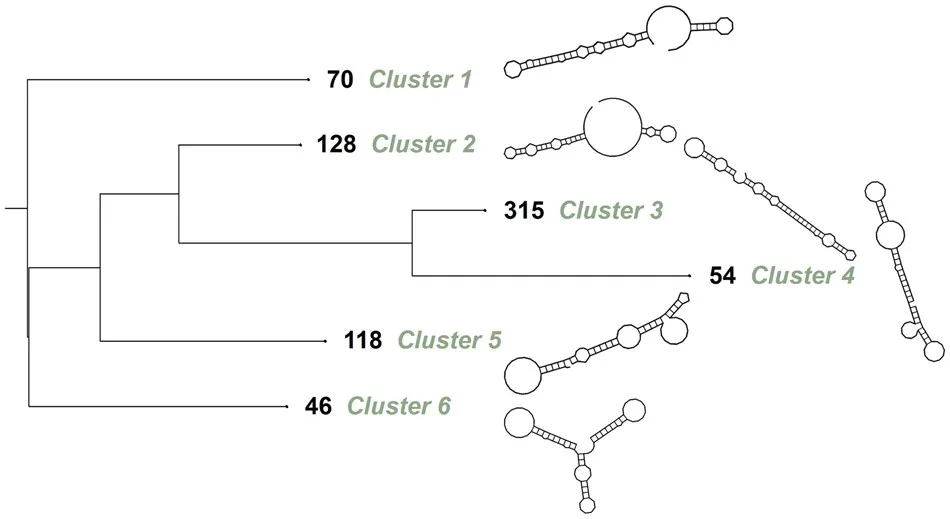
The phylogenetic tree showing the cluster representatives’ secondary structure.

#### 3.3.2 Second approach: separate analysis of enriched sequences in the positive and negative selection pool

##### 3.3.2.1 Clustering and multiple sequence alignment of the preprocessed sequencing data from the positive and negative selection pool

In the second approach, enriched sequences from the positive and negative selection pools were analyzed independently. As in the first approach, MSA was performed, sequence homologies were assessed via phylogenetic tree analysis, and secondary structures were compared. For downstream analyses, only clusters containing more than 10 members were considered. The positive selection pool yielded 7 clusters, whereas the negative selection pool resulted in 6 distinct clusters (Table 2). Among the positive pool, aptamer candidates 1 188 996, 371 593, and 227 207, belonging to Clusters 3, 5, and 4, respectively, exhibited strong enrichment.

**Table 2:** Clusters of positive and negative selection pool.

Cluster Nr.	Sequence numbers in the *positive* pool	Cluster Nr.	Sequence numbers in the *negative* pool
1	19	8	270
2	1704	9	624672
3	1188996	10	7230
4	227207	11	217629
5	371593	12	72728
6	24770	13	63864
7	82234	–	–

##### 3.3.2.2 Identification of secondary structures and reconstruction of the phylogenetic tree

Phylogenetic tree analysis, based on primary sequence homology, clustered the candidate aptamers from the positive and negative selection pools according to their potential evolutionary relationships. The genetic distance is indicated by branch length, reflecting the degree of similarity between sequences. Internal nodes identify common ancestors so that closely related sequences can be identified by examining their most recent common ancestor. Clusters 1, 2, 4, and 5 from the positive selection pool emerged as the most promising candidates, as their sequences showed minimal similarity to clusters derived from the negative selection pool (Fig. 5).

**Figure 5:**
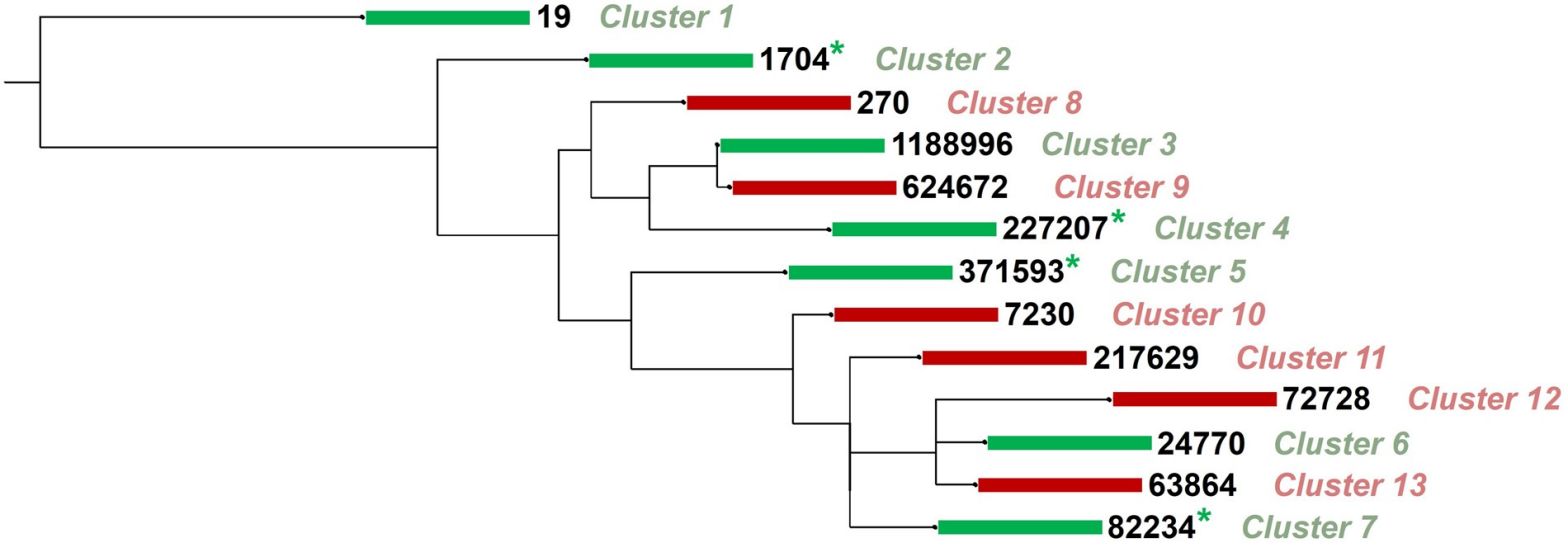
Phylogenetic tree of cluster representatives after applying an abundance filter with a minimum threshold of 10 members. In total, 13 distinct clusters were identified. Clusters of the positive selection pool are visualized in green, and clusters of the negative selection pool in red. Asterisks (*) indicate the selected clusters.

Using ViennaRNA, the secondary structure of a representative aptamer from each cluster was predicted (Fig. 6). The sequence 1188996 (Cluster 3) from the positive selection pool and the sequence 624672 (Cluster 9) from the negative selection pool exhibited structural similarity (Fig. 6, dotted box), consistent with their close relationship observed in the previous phylogenetic analysis (Fig. 5). Although phylogenetic analysis indicated that the aptamer sequence 1704 from Cluster 2 could be a suitable candidate, its secondary structure was too similar to that of the sequence 624 672 (Cluster 9) from the negative selection pool. Thus, among the six aptamer sequences from the positive selection pool representing each cluster, the aptamers 82 234 (Cluster 7), 371 593 (Cluster 5), and 227 207 (Cluster 4) were selected for further validation due to their abundance and large genetic distance from aptamer sequences of the negative selection pool.

**Figure 6:**
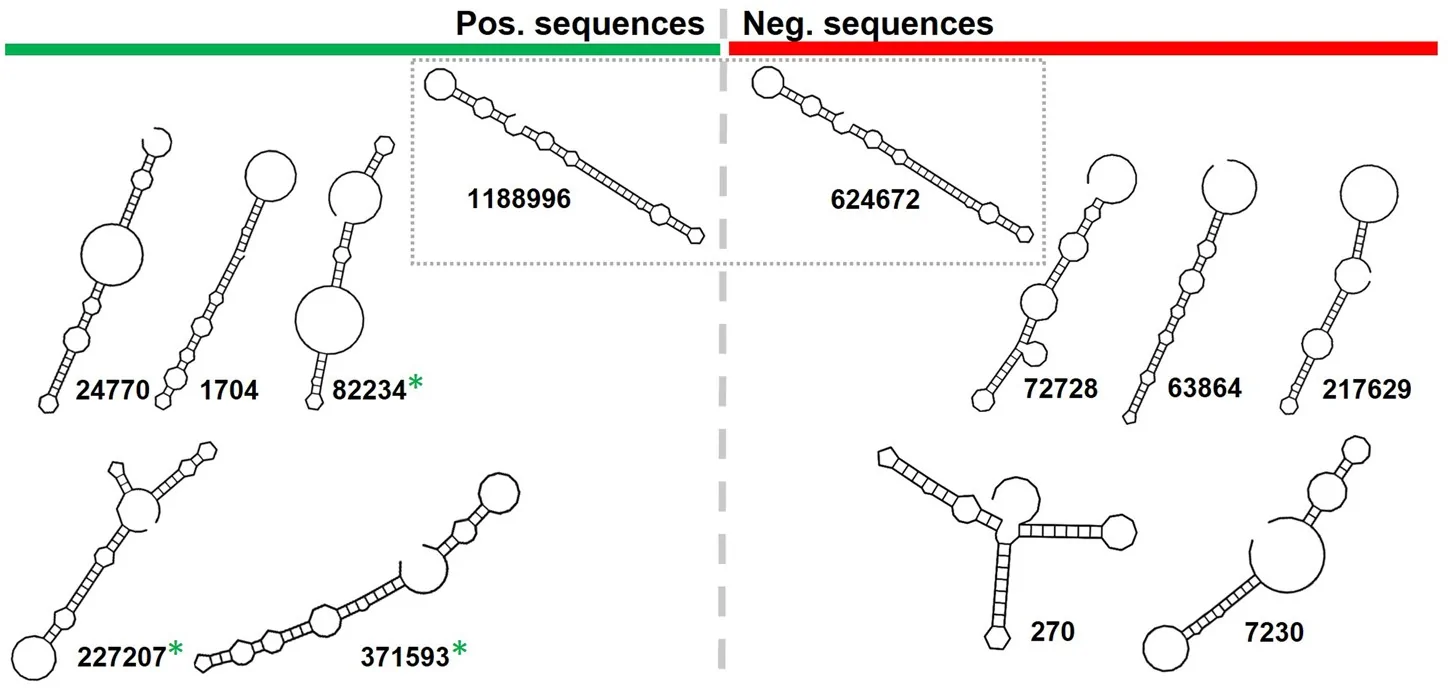
Secondary structure analysis of the aptamer sequences for the identification of structural motifs. The sequences illustrated within the dotted grey box are structurally very similar. Asterisks (*) indicate the selected aptamers.

### 3.4 Analysis of the selective binding of the candidate aptamers to the target protein

Dot blot analysis was conducted to evaluate the binding of the selected biotinylated aptamers to the target and nontarget protein (Fig. 7A). All selected aptamers demonstrated binding to the target protein, whereas no binding or only minimal binding was detected to the nontarget protein, confirming the specificity of the selected aptamers. The quantification of the dot blot signals revealed that aptamer 128 exhibited the highest signal intensity among all tested aptamers (Fig. 7B), indicating stronger binding to the target protein. Overall, these results validated the efficiency and successful applicability of the developed aptamer selection procedure.

**Figure 7:**
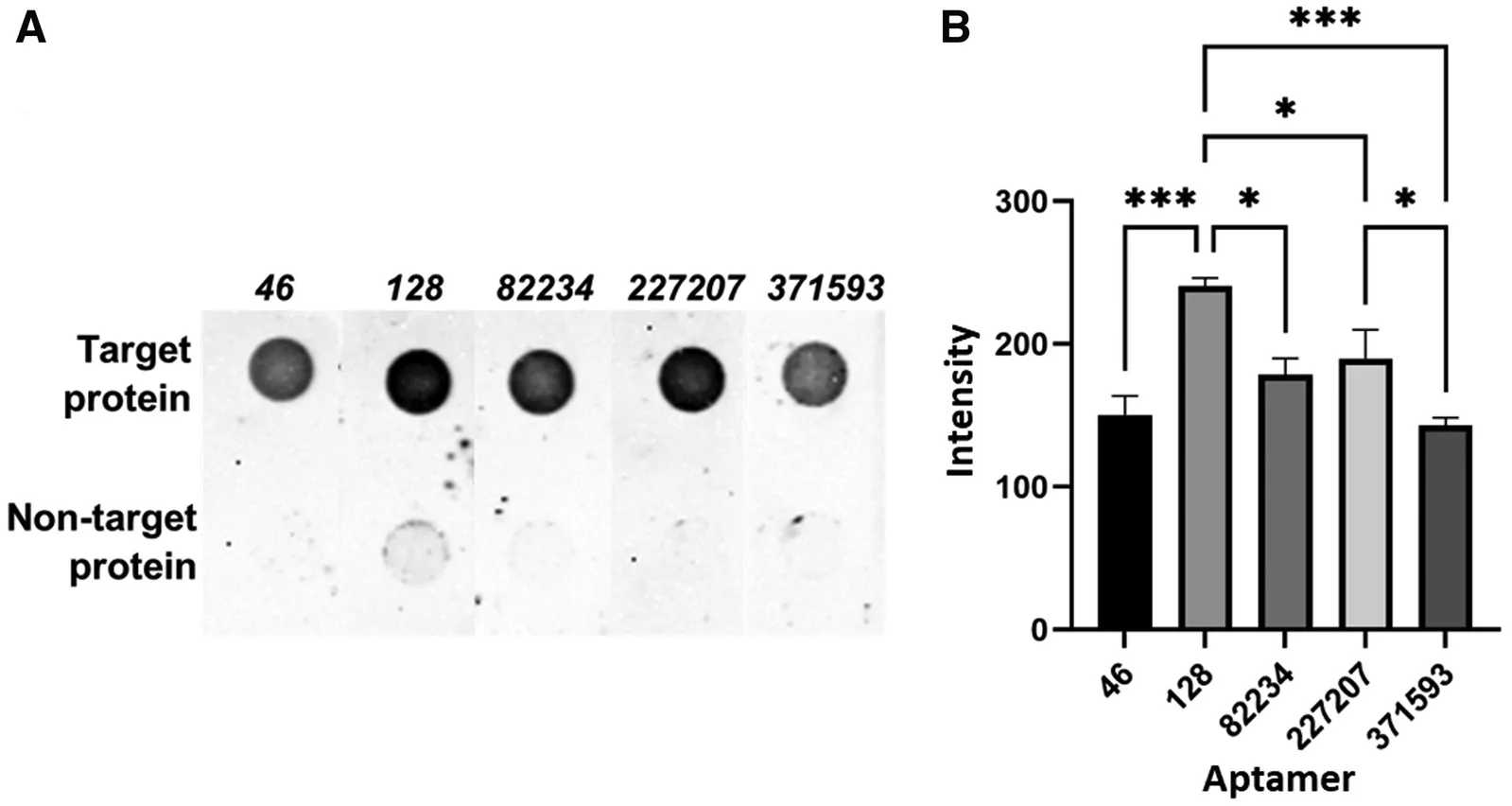
(A) Analysis of aptamer binding using dot blot assay. To perform the analysis, 10 µg of target protein or nontarget protein was immobilized on the membrane and incubated with 80 pmol of each selected biotinylated aptamer. (B) Quantification of dot blot signals. Chemiluminescence signal intensities were quantified using ImageJ and are presented as 8-bit grayscale mean intensity values. Statistical significance was determined by one-way ANOVA followed by Tukey’s multiple comparisons test. *P ≤ .05, ***P ≤ .001.

## 4 Discussion

Previously, only a few hundred clones from enriched DNA pools were sequenced via Sanger sequencing to identify potent aptamers, whereas today, NGS enables the simultaneous analysis of millions of sequences (Yüce *et al.* 2015). Among NGS platforms, Illumina sequencing platform can be used in SELEX, primarily due to its high throughput and longer read lengths, which provide more comprehensive coverage of aptamer pools (Schütze *et al.* 2011). However, despite these advantages, the large volume of NGS data and the complexity of associated workflows present significant challenges. The full potential of NGS often remains underexploited, largely due to the demanding data processing requirements, the need for substantial bioinformatic expertise, and the limited availability of user-friendly analysis tools (Zhu *et al.* 2019). This represents a persistent bottleneck in SELEX with NGS workflows: without sufficiently advanced computational tools, valuable sequence patterns and enrichment dynamics may remain obscured. Robust and dedicated bioinformatic workflows are therefore essential for the effective processing, interpretation, and utilization of complex NGS-derived SELEX datasets.

In this work, we aimed to demonstrate how a comprehensive NGS analysis of the SELEX-evolved DNA pools can be employed to select the target-binding aptamers. Integrating NGS with bioinformatic analysis can enable improved identification of aptamers, optimizing the selection of sequences with high affinity and specificity. A negative selection was performed using a nontarget protein to identify sequences enriched specifically for the target. To ensure compatibility with the Illumina platform, sequencing-ready libraries were generated through a two-step PCR that introduced the required adapter sequences. Following NGS, comprehensive data preprocessing and computational analysis were conducted to extract potent candidates from the enriched aptamer pool.

Although NGS has become widely adopted for the identification of enriched aptamers after SELEX (Komarova *et al.* 2020), the extent and transparency of the associated computational analyses differ considerably among published studies. Previous studies have identified candidate aptamers primarily based on sequence abundance while providing limited information regarding preprocessing, quality filtering, clustering strategies, or downstream analyses (Kupakuwana *et al.* 2011, Oliveira *et al.* 2024). Similarly, some workflows have prioritized candidate sequences primarily on the basis of their enrichment without incorporating information from the negative selection pool or phylogenetic analyses (Yamamoto *et al.* 2023). Other studies have demonstrated that the most abundant sequence is not necessarily the aptamer with the highest binding affinity (Lim *et al.* 2022). Collectively, these findings highlight the limitations of abundance-based candidate selection and underscore the need for comprehensive and reproducible analytical workflows that integrate multiple complementary criteria for reliable candidate prioritization.

Sequence abundance is commonly used as a primary criterion for selecting candidate binders based on the assumption that target-specific binders progressively accumulate during successive SELEX rounds. However, sequence enrichment alone does not always correlate with target-binding affinity, and high-affinity binders may remain underrepresented in the selected pool (Spiga *et al.* 2015). Some sequences may become highly abundant despite exhibiting weak binding, primarily due to biases introduced during PCR amplification. Amplification efficiency can vary among sequences depending on factors such as GC content, secondary structure, and sequence motifs (Levay *et al.* 2015). Consequently, sequences that amplify more efficiently can become disproportionally enriched, whereas sequences that form stable secondary structures or fold into strong target-binding conformations may be amplified less efficiently (Zimmermann *et al.* 2010, Schütze *et al.* 2011). Such biases can become particularly pronounced when a high number of PCR cycles is used, as even small differences in amplification efficiency can be exponentially amplified over successive cycles. Therefore, relying solely on sequence abundance can be misleading, and additional criteria, such as conserved motifs and predicted secondary structures, are essential to more accurately prioritize candidate aptamers for subsequent functional validation.

To address these limitations, we applied two complementary computational strategies to identify aptamer candidates and incorporated analyses of their predicted secondary structures and phylogenetic relationships to assess sequence similarities. The first approach focused on subtracting sequences present in the negative selection pool from those in the positive pool. MSA and phylogenetic clustering were then used to efficiently group dereplicated sequences into sequence families (clusters). Furthermore, consensus motifs and predicted secondary structures were evaluated. Aptamers frequently adopt complex structures, including stem-loops, G-quadruplexes, and pseudoknots, highlighting the critical role of structural context in candidate prioritization (Ochoa and Milam 2025). Consequently, enriched sequences were selected not only based on their abundance but also on computational predictions of secondary structure. Six major clusters were identified, and two representatives with the most promising predicted structures and enrichment profiles were chosen for binding validation.

Although subtractive filtering against the negative selection pool can enhance specificity, it also reduced cluster abundance, suggesting that some potentially relevant candidates may be lost through strict exclusion. To address this limitation, a second analytical strategy was employed in which abundance levels were compared between the positive and negative pools without discarding sequences shared between them. Here, the focus was on aptamers with a high abundance difference between the positive and negative pools, preferentially with a high abundance in the positive pool. This approach revealed seven clusters in the positive selection pool and six in the negative selection pool. Phylogenetic reconstruction further enabled the identification of sequences with minimal relatedness to those present in the negative selection pool. Among these, four candidates exhibited weak similarity to sequences of the negative selection pool. Three candidates from the positive selection pool were ultimately selected for further experimental validation based on an integrated combination of abundance, sequence characteristics, and predicted secondary structures.

Dot blot analysis of all five aptamer candidates identified through the two analytical strategies confirmed specific binding to the target protein compared to the nontarget protein, with two aptamer candidates originating from the first approach (subtracting sequences of the negative selection pool from the positive selection pool) and three being selected through the second approach (separate analysis of enriched sequences in the positive and negative selection pools). Candidates selected through either strategy exhibited comparable binding behaviors, demonstrating the robustness and complementarity of both analytical workflows. Quantitative analysis of the dot blot signals showed that aptamer 128, which was identified using the first approach, exhibited the highest binding signal among the analyzed aptamers and showed no detectable binding to the nontarget protein. Overall, both strategies successfully identified aptamers with specific target binding and can therefore be considered complementary rather than competing approaches. The choice of workflow may depend on the specific objectives of the study and the characteristics of the sequencing dataset.

Compared to Sanger sequencing, the NGS-based approach does not require the prior selection of individual clones and enables access to the full diversity of the selected sequence pool. Given that Sanger-based methods are restricted to the analysis of a limited number of sequences, they may overlook rare but high-affinity binders that can be more readily captured through comprehensive NGS analysis. Overall, these findings highlight that NGS-guided SELEX augmented by systematic bioinformatic processing effectively identifies aptamers with strong and selective binding characteristics.

## 5 Conclusion

This work presents a comprehensive and systematic workflow that integrates SELEX, NGS, and advanced bioinformatic analysis to streamline the discovery of target-specific aptamers. By either subtracting sequences from the negative selection pool from those in the positive selection pool or by separately analyzing both pools and search for enriched sequences in the positive pool in combination with clustering, phylogenetic grouping, and structural evaluation, we demonstrated that aptamers with specific target binding properties can be reliably obtained. The successful binding validation of selected candidates using dot blot analysis confirmed the applicability and reliability of the established analysis strategy.

In conclusion, the methodology described here provides a practical and scalable framework for aptamer discovery, facilitating the transition from large and diverse sequence pools to a focused set of high-quality candidates suitable for downstream characterization and potential application in research, therapy, and diagnostic settings.

## Data Availability

The data underlying this article will be shared on reasonable request to the corresponding author.
